# Chidamide plus envafolimab as subsequent treatment in advanced non‐small cell lung cancer patients resistant to anti‐PD‐1 therapy: A multicohort, open‐label, phase II trial with biomarker analysis

**DOI:** 10.1002/cam4.7175

**Published:** 2024-04-10

**Authors:** Yaxiong Zhang, Zihong Chen, Yu Liu, Liang Han, Wei Jiang, Qiming Wang, Jianhua Shi, Liqin Lu, Jianying Li, Mingjun Zhang, Yan Huang, Yunpeng Yang, Xue Hou, Li Zhang, Jing Li, Wenfeng Fang, Gang Chen

**Affiliations:** ^1^ Department of Medical Oncology, State Key Laboratory of Oncology in South China, Guangdong Provincial Clinical Research Center for Cancer, Collaborative Innovation Center for Cancer Medicine Sun Yat‐sen University Cancer Center Guangzhou China; ^2^ Zhongshan School of Medicine Sun Yat‐sen University Guangzhou China; ^3^ Department of Clinical Research, State Key Laboratory of Oncology in South China, Guangdong Provincial Clinical Research Center for Cancer, Collaborative Innovation Center for Cancer Medicine Sun Yat‐sen University Cancer Center Guangzhou China; ^4^ Department of Oncology Xuzhou Central Hospital Xuzhou Jiangsu China; ^5^ Department of Respiratory Oncology Guangxi Medical University Cancer Hospital Nanning Guangxi China; ^6^ Department of Internal Medicine, Henan Cancer Hospital Affiliated Cancer Hospital of Zhengzhou University Zhengzhou Henan China; ^7^ Department of Oncology Linyi Cancer Hospital Linyi Shandong China; ^8^ Department of Medical Oncology The People's Hospital of Zhejiang Province Hangzhou Zhejiang China; ^9^ Department of Oncology Nantong Tumor Hospital Nantong Jiangsu China; ^10^ Department of Oncology The Second Hospital of Anhui Medical University Hefei Anhui China; ^11^ State Key Laboratory of Oncology in South China, Guangdong Provincial Clinical Research Center for Cancer, Collaborative Innovation Center for Cancer Medicine Sun Yat‐sen University Cancer Center Guangzhou China

**Keywords:** chidamide, envafolimab, non‐small cell lung cancer, PD‐1/PD‐L1, resistant

## Abstract

**Background:**

Combination of chidamide and anti‐PD‐L1 inhibitor produce synergistic anti‐tumor effect in advanced NSCLC patients resistant to anti‐PD‐1 treatment. However, the effect of chidamide plus envafolimab has not been reported.

**Aims:**

This study aimed to evaluate the efficacy of chidamide plus envafolimab in advanced NSCLC patients resistant toanti‐PD‐1 treatment.

**Materials and Methods:**

Eligible advanced NSCLC patients after resistant to anti‐PD‐1 therapy received chidamide and envafolimab. The primary endpoint was objective response rate (ORR). The secondary end points included disease control rate (DCR), progression‐free survival (PFS), and safety. The expression of histone deacetylase 2 (HDAC2), PD‐L1, and blood TMB (bTMB) was also analyzed.

**Results:**

After a median follow‐up of 8.1 (range: 7.6–9.2) months, only two patients achieved partial response. The ORR was 6.7% (2/30), DCR was 50% (15/30), and median PFS (mPFS) was 3.5 (95% confidence interval: 1.9–5.5) months. Biomarker analysis revealed that patients with high‐level HDAC2 expression had numerically superior ORR (4.3% vs. 0), DCR (52.2% vs. 0) and mPFS (3.7 vs. 1.4m). Patients with negative PD‐L1 had numerically superior DCR (52.2% vs. 33.3%) and mPFS (3.7m vs. 1.8m), so were those with low‐level bTMB (DCR: 59.1% vs. 16.7%, mPFS: 3.8 vs.1.9m). Overall safety was controllable.

**Discussion:**

High HDAC2patients showed better ORR, DCR, and PFS. In addition, patient with negative PD‐L1 and low‐level bTMB had better DCR and PFS. This may be related to the epigenetic function of chidamide. However, the sample size was not big enough, so it is necessary to increase sample size to confirm the conclusion.

**Conclusion:**

Combination of chidamide and envafolimab showed efficacy signals in certain NSCLC patients. But further identification of beneficial population is necessary for precision treatment.

## 1 INTRODUCTION

Approximately 85% of lung cancer are non‐small cell lung cancer (NSCLC).^1^ However, 70% of these patients were diagnosed with advanced stage at their initial visit,^2^, ^3^ losing the opportunity of radical operation. These patients can only turn to traditional chemoradiotherapy. Platinum‐based double‐drug chemotherapy was a classic regimen for patients with advanced NSCLC who lack driver mutation, with median progression‐free survival (mPFS) and median overall survival of 5–6 months and 11–12 months, respectively.^4^ Long term efficacy for chemotherapy is poor. After a long period of exploration, immunotherapy gradually matured and is now demonstrating its efficacy.^5^ Its immune check point inhibitors (ICIs) such as programmed death‐1 (PD‐1)/programmed death ligand‐1 (PD‐L1) inhibitors are attracting much attention. Immunotherapy restores antitumor activity of effector T‐cells by blocking PD‐1/PD‐L1 signal pathway to control tumor.^6^

Nowadays, PD‐1/PD‐L1 ICIs monotherapy or in combination with chemotherapy has been approved as standard treatment for advanced NSCLC without driver mutation. Objective response rate (ORR) for first‐line PD‐1/PD‐L1 ICIs in combination with platinum‐based chemotherapy is about 50%. While for second‐line ICIs monotherapy, ORR is about 15%–20%.^7^ However, disease progression (PD) usually develops within 1 year due to secondary resistance.^8^ Guidelines of National Comprehensive Cancer Network consider that another PD‐1/PD‐L1 ICI is not recommended when PD‐1/PD‐L1 ICI therapy is failure.^9^ Thus, post‐line treatment is limited to single‐drug chemotherapy,^10^ with an ORR of about 10%.^11^ Despite such a huge patient population, there is still no effective treatment. How to deal with advanced NSCLC patients when they are resistant to PD‐1/PD‐L1 inhibitors has become an urgent challenge.

Chidamide, an epigenetic regulatory drug, is the only orally administered subtype‐selective histone deacetylase (HDAC) inhibitor that regulates host immunity and tumor microenvironment and has a potential role in restoring resistance sensitivity as well as inhibiting metastasis and recurrence.^12^ In a phase I study in which 31 patients with advanced solid tumors and lymphomas, no DLTs were identified in the BIW cohorts up to 50 mg. Out of the 25 evaluable patients, there were five patients with partial response (PR), 11 patients with stable disease (SD).^13^ Chidamide was generally well tolerated and encouraging preliminary anti‐tumor activity was demonstrated. It has been approved as treatment for recurrent or refractory peripheral T‐cell lymphoma and some breast cancer patients.^14^, ^15^

Envafolimab is the first subcutaneous anti‐PD‐L1 antibody in the worldwide which specifically binds to PD‐L1 to blocks interaction of PD‐1 and PD‐L1.^16^ It has accumulated a large amount of safety data through clinical research.^17^ In previous phase I studies, no DLTs were reported in patients with advanced solid tumors who received envafolimab up to 300 mg Q4W. And ORR was 10.7%–11.4%. Disease control rate (DCR) was 34.3%–39.3%.^18^, ^19^ Envafolimab is currently approved for the treatment of advanced solid tumors with unresectable or metastatic microsatellite instability‐high or deficiency mismatch repair.^20^

Preclinical studies have demonstrated that chidamide in combination with PD‐L1 inhibitors produce synergistic tumor suppressive effect.^21^ Combination therapy continues to produce antitumor effects after PD of anti‐PD‐1 treatment, suggesting that combination therapy has the potential to prevent primary resistance and reverse secondary resistance in PD‐1 inhibitor therapy.^22^ In summary, it is worth conducting clinical trials of chidamide plus envafolimab in advanced NSCLC patients who were resistant to PD‐1 inhibitors to explore the initial efficacy and safety of combination therapy.

## 2 METHOD

### 2.1 Patients

This study recruited patients from 12 centers in China between August 2021 and November 2022. Patients with histologically or cytologically confirmed unresectable stage IIIB‐IV NSCLC after PD of anti‐PD‐1 treatment could be enrolled. According to the Society for Immunotherapy of Cancer (SITC), resistance to ICIs can be classified into primary resistance and secondary resistance.^23^ Primary resistance was defined that best overall response (BOR) is PD or duration of SD is less than 6 months during anti‐PD‐1 treatment (at least 6 weeks or two treatment cycles).^24^ Secondary resistance was defined that BOR is complete response (CR) or PR, or duration of SD is at least 6 months. However, PD was still developed later in the treatment (including within 3 months of last PD‐1 inhibitor medication).^24^ Additional inclusion criteria included: age 18 years or older, previously received less than or equal to three lines of systemic chemotherapy, without EGFR/ALK/ROS1/RET mutations, Eastern Cooperative Oncology Group (ECOG) performance status (PS) 0–1, sufficient organ function, and measurable disease according to the Response Evaluation Criteria in Solid Tumors 1.1 (RECIST 1.1). The critical exclusion criteria consisted of previously receiving anti‐PD‐L1/anti‐PD‐L2/anti‐CTLA‐4 treatment, previously using HDAC inhibitor (such as chidamide), preexisting autoimmune diseases, and ongoing steroid treatment with prednisone >10 mg/day orally or equivalent. The trial was approved by the Ethics Committee of Sun Yat‐sen University Cancer Center and conducted in accordance with the Declaration of Helsinki and Good Clinical Practice.

### 2.2 Treatment

The study was an explored, open‐label, phase II trial conducted in a dose escalation clinical setting. The treatment cycle was 4 weeks. All enrolled patients should receive envafolimab 400 mg every 4 weeks by hypodermic injection in hospital. In the first stage, three patients were enrolled to receive envafolimab 400 mg every 4 weeks plus chidamide 20 mg p.o. twice a week (the interval should not be less than 3 days). Dose of chidamide would be escalated to 30 mg p.o. twice a week if less than or equal to one patient experienced dose‐limiting toxicity in the first stage and additional patients were enrolled at that level in an expansion cohort.

### 2.3 Assessment

Tumor response was assessed by investigators per RECIST 1.1 every 8 weeks (±7 days). Patients with first radiologic evidence of PD would continue treatment until the investigators estimated that they would not get benefit from later treatment. After the first 30 patients in the second stage completed at least one post‐baseline efficacy evaluation, the interim analysis would be carried out.

### 2.4 Endpoint

The primary end point was ORR. The secondary end points included DCR, PFS, and OS. PFS was defined as the time from the first dose of either drug to investigator assessed radiologic PD or to death of any cause. PFS would be censored at the time of last tumor assessment. OS was defined as the time from patient enrollment to death of any cause. Safety was assessed throughout the study. Adverse events (AEs) were graded according to the Common Terminology Criteria for Adverse Events (version 5.0).

### 2.5 Biomarker analysis

PD‐L1 expression was analyzed by immunohistochemistry of paraffin‐embedded tumor samples. PD‐L1 expression was measured by Ventana PD‐L1 (SP263) assay from Roche (Medx Translation Medicine, Suzhou, CHN), and positive PD‐L1 was defined as percentage of tumor cell of with any membrane staining above background (TC%) greater than or equal to 1%.

Blood samples would also be collected at baseline for subsequent tumor mutation burden (TMB) assessment. The blood TMB (bTMB) was measured by YuceOne Plus (ctDNA) targeting 1012 genes (Yukang, Shenzhen, CHN). For the calculation of the bTMB, we applied three criteria for competent mutations: (1) somatic but not germline mutation; (2) located in the coding region, nonsynonymous SNVs/Indels; and (3) a mutation allele frequency ≥0.5%. The bTMB was defined as the number of competent mutations per megabase on the exome examined. High‐level bTMB was defined as greater than 10 mutations per Mb.

HDAC2 expression was analyzed by the immunohistochemistry of formalin‐fixed paraffin‐embedded tumor samples on an automatic immunochemistry staining machine (Lecia Bond RX, 1:4500; ab219053; Abcam, Cambridge, UK). Tumor samples combined with recombination anti‐HDAC2 antibody (EPR20117, Abcam, Cambridge, UK) first, then combined with second antibody which was marked by horseradish peroxidase. Finally, EnVision FLEX+ DAB substrate kit (DAKO K800221‐2, Agilent, California, US) made HDAC2 positive area brown. The testing company was Shenzhen Yukang Medical Testing Laboratory. Once the nucleus was staining for any degree, we thought that cell was positively staining. If the staining was weak, we recorded 1+. If weak to medium, then 2+. If strong and uniform, we recorded 3+. When positive cell proportion was 5% or higher, then an “H‐score” of tumor cells should be made. H‐score = [1 × (% cells 1+) + 2 × (% cells 2+) + 3 × (% cells 3+)]. High‐level HDAC2 expression was defined as H‐score equaled to 300. Medium‐level HDAC2 expression was defined as H‐score equaled to 200. While low‐level HDAC2 expression was defined as H‐score equaled to 80.

Gene expression data for gene expression profile was generated by extracting RNA of tumor samples, removing ribosomes, and construcing a whole transcriptome library of total RNA. The sequencing libraries were prepared with NadPrep DNA Library Preparation Module for MGI (Nanodigmbio, CHN) according to the manufacturer's instructions and sequenced on the DNBSEQ‐T7 sequencer (BGI, CHN) with 100 bp paired‐end reads following standard procedures. Detailed genes were listed on Table S1.

### 2.6 Statistical analysis

The previously reported ORR of single‐drug chemotherapy was about 10%.^11^ We expected an ORR of 24% for combination treatment. The sample size of 56 patients could provide at least 90% power to detect the treatment effect different from previously reported ORR (two‐sided *α*‐value of 0.1). Considering 10% drop‐out rate, 63 patients would be enrolled. The primary and secondary end points were analyzed for the per protocol set, while the AEs were analyzed for the safety set (SS). The ORR and DCR analyses were based on frequencies calculated with corresponding two‐sided 95% confidence intervals (CIs) using the Clopper–Pearson method. The Kaplan–Meier method was used for the analysis of PFS and OS. All statistical analyses were performed using SAS software (version 9.4).

Fastp (version 0.19.5) was adopted to remove adapters and low‐quality RNA‐seq reads. Then, the clean reads were mapped to the human reference genome (version hg38/GRCh38) using STAR v.2.7.8a. Reads counts of each transcriptome data were calculated using FeatureCount (Version 1.5.2). Fragments Per Kilobase per million (FPKM) was used to normalize the count data and then log10 transformed (log10 (FPKM+1)).

DESeq2 R package was used for gene differential expression analysis between the two groups. The genes with |log2 fold change (log2FC)| >1 and adjusted *p*‐value <0.05 were considered as differential expression genes (DEGs). Gene Ontology biological processes and Kyoto Encyclopedia of Genes and Genomes (KEGG) pathway enrichment analysis of DEGs were performed by ClusterProfiler R package.

## 3 RESULTS

### 3.1 Patient enrollment and baseline characteristics

**FIGURE 1:**
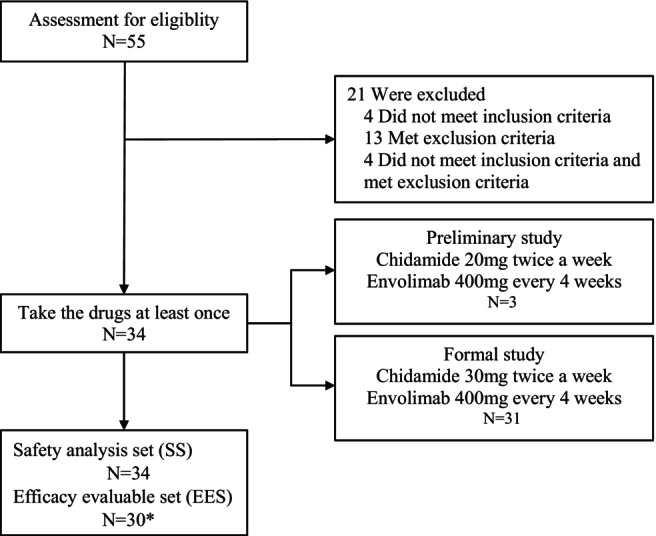
Eligibility and analysis. *Four patients lacked of efficacy evaluation after treatment, so only 30 patients were included in the efficacy evaluable set.

Between August 2021 and November 2022, a total of 55 patients were assessed for eligibility from 12 centers in China. Among them, 34 patients were included and received at least one dose of study treatment. All of them were included in the safety analysis, but only 30 patients were included in the efficacy analysis since four cases lacked of efficacy evaluation after treatment (Figure 1). Patients were predominantly men (91.2%, 31/34), with an ECOG PS score of 1 (94.1%, 32/34) and diagonosed with stage IV (91.2%, 31/34). A total of 14 patients (41.2%, 14/34) had squamous cell carcinoma, 17 patients (50.0%, 17/34) had adenocarcinoma. For biomarker analysis, four were positive PD‐L1 (11.8%, 4/34), nine were high‐level bTMB (26.5%, 9/34), and 26 were high‐level HDAC2. 32 patients (94.1%, 32/34) had previously received PD‐1 inhibitor combination chemotherapy. Nine (26.5%, 9/34) were primary resistant, and 20 (58.8%, 20/34) were secondary resistance. The detailed baseline characteristics of the participants were illustrated in Table 1. At the time of data cutoff (February 23, 2023), the median follow‐up time was 8.1 (range: 7.6–9.2) months.

**TABLE 1:** Baseline patient characteristics.^a^

Characteristics	Chidamide plus envafolimab (*N* = 34)
Median age, years (range)	59.0 (27–74)
Sex	
Male	31 (91.2)
Female	3 (8.8)
Smoking history	
Never‐smoker	6 (17.6)
Ex‐smoker	24 (70.6)
Smoker	4 (11.8)
ECOG PS	
0	2 (5.9)
1	32 (94.1)
Histology type	
Squamous	14 (41.2)
Adenocarcima	17 (50.0)
Others^b^	3 (8.8)
Stage^c^	
IIIB/IIIC	3 (8.8)
IV	31 (91.2)
Brain metastasis	
No	32 (94.1)
Yes	2 (5.9)
PD‐L1 expression	
Negative (<1%)	25 (73.5)
Positive (≥1%)	4 (11.8)
1%–49%	4 (11.8)
≥50%	0
NE	5 (14.7)
bTMB expression	
High (>10Muts/Mb)	9 (26.5)
Low (≤10Muts/Mb)	23 (67.6)
NE	2 (5.9)
HDAC2 expression	
High	26 (76.5)
Low/medium	2 (5.9)
NE	6 (17.6)
Lines of previous NSCLC therapy	
1	20 (58.8)
2	11 (32.4)
≥3	3 (8.8)
Previous treatment of PD‐1 inhibitor	
Monotherapy	2 (5.9)
Combination	32 (94.1)
Resistant type to previous PD‐1 inhibitor treatment	
Primary resistance	9 (26.5)
Secondary resistance	20 (58.8)
NE	5 (14.7)

^a^ Data are No. (%).

^b^ Other histological type includes adenosquamous, lymphoepithelioma‐like carcinoma, and unknown.

^c^ Defined by American Joint Committee on Cancer, eighth edition.

Abbreviations: bTMB, blood tumor mutation burden; ECOG PS, Eastern Cooperative Oncology Group performance status; HDAC2, histone deacetylase 2; Muts/Mb, mutations per Mb; NE, not evaluable; NSCLC, non‐small cell lung cancer; PD‐L1, programmed death‐ligand 1.

### 3.2 Efficacy

**FIGURE 2:**
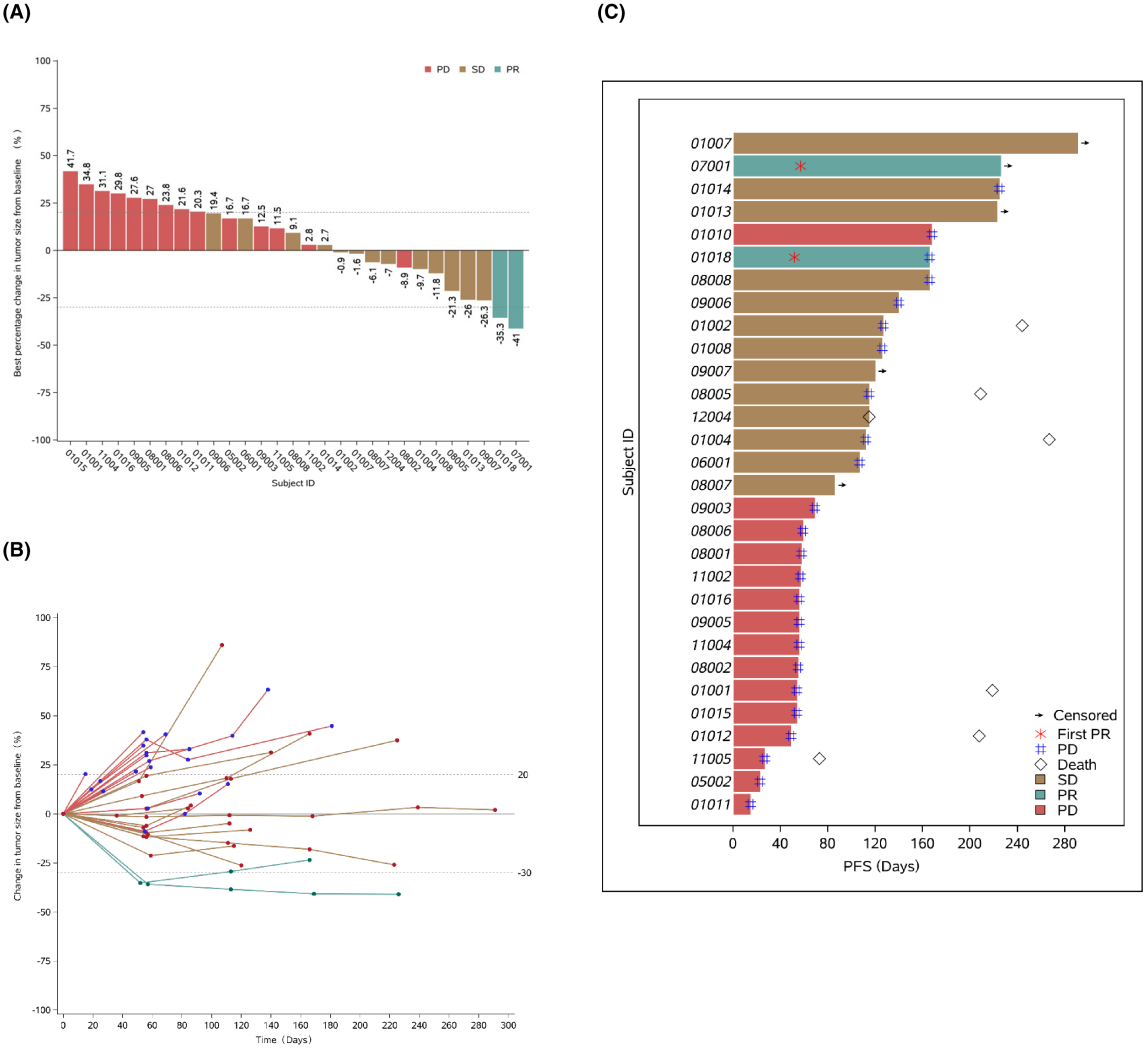
Objective response rate and progression‐free survival (PFS). (A) Objective response rate of each patient. (B) Change of tumor size overall time. (C) Progression‐free survival. Four cases (patient ID: 05001, 11003, 12002, and 12003) lack of efficacy evaluation after treatment. Target lesion of one patient (patient ID: 01010) cannot be evaluated, but overall evaluation was PD due to appearance of new lesions. Evaluation for target lesions of five patients (patient ID: 05002, 08002, 09003, 11002, and 11005) was stable disease (SD), overall evaluation was PD due to appearance of new lesions. PR, partial response.

**FIGURE 3:**
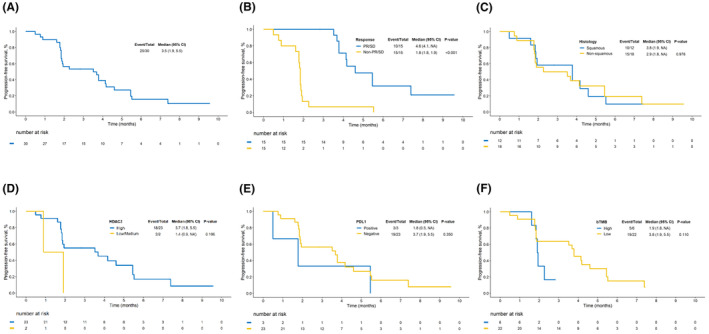
Progression‐free survival (A) efficacy evaluation set population. (B) Responsive (PR/SD) versus irresponsive. (C) Squamous versus non‐squamous. (D) By histone deacetylase 2 (HDAC2) expression (high vs. low/medium). (E) By programmed death ligand 1 (PD‐L1) status (positive vs. negative). (F) By blood tumor mutation burden (bTMB) status (high vs. low). PR, partial response; SD, stable disease.

The ORR was 6.7% (2/30) and DCR was 50% (15/30) (Table 2, Figure 2A,B). PFS events occurred in 25 patients and the mPFS was 3.5 months (95% CI: 1.9%–5.5%) (Figures 2C and 3A). In sub‐group analysis, responsive group (BOR is PR/SD) had a longer mPFS (4.6 m vs. 1.8 m, *p*‐value <0.001) (Figure 3B). The benefit of chidamide plus envafolimab was regardless of histological type, in terms of ORR (squamous vs. non‐squamous, 8.3% vs. 5.6%, *p* = 1.000) and DCR (squamous vs. non‐squamous, 50.0% vs. 50.0%, *p* = 1.000) (Table 2). PFS was similar between patients with squamous cell carcinoma and with non‐squamous cell carcinoma (mPFS: 2.9 m vs. 3.8 m, *p* = 0.976) (Figure 3C). No statistically significant difference was found in ORR (10.5% vs. 0, *p* = 0.520), DCR (52.6% vs. 45.5%, *p* = 1.000) and mPFS (3.7 m vs. 3.5 m, *p* = 0.900) between patients who received prior first line of treatment and those who received at least two lines of treatment. ORR (0 vs. 10.5%, *p* = 1.000), DCR (50.0% vs. 52.6%, *p* = 1.000) and mPFS (4.1 m vs. 3.5 m, *p* = 0.639) were all similar between patients with primary resistance and secondary resistance (Figure S1).

**TABLE 2:** Tumor response.^a^

EES	Overall (*n* = 30)	Squamous (*N* = 12)	Non‐squamous (*N* = 18)	High HDAC2 (*N* = 23)	Low/medium HDAC2 (*N* = 2)	Positive PD‐L1 (*N* = 3)	Negative PD‐L1 (*n* = 23)	High bTMB (*N* = 6)	Low bTMB (*N* = 22)
BOR, *n* (%)									
CR	0	0	0	0	0	0	0	0	0
PR	2 (6.7)	1 (8.3)	1 (5.6)	1 (4.3)	0	1 (33.3)	0	0	2 (9.1)
SD	13 (43.3)	5 (41.7)	8 (44.4)	11 (47.8)	0	0	12 (52.2)	1 (16.7)	11 (50.0)
PD	15 (50.0)	6 (50.0)	9 (50.0)	11 (47.8)	2 (100.0)	2 (66.7)	11 (47.8)	5 (83.3)	9 (40.9)
NE	0	0	0	0	0	0	0	0	0
ORR, *n* (%)	2 (6.7)	1 (8.3)	1 (5.6)	1 (4.3)	0	1 (33.3)	0	0	2 (9.1)
95% CI	(0.8–22.1)	(0.2–38.5)	(0.1–27.3)	(0.1–21.9)		(0.8–90.6)			(1.1–29.2)
*p*‐value		1.000		1.000		0.115		1.000	
DCR, *n* (%)	15 (50.0)	6 (50.0)	9 (50.0)	12 (52.2)	0	1 (33.3)	12 (52.2)	1 (16.7)	13 (59.1)
95%CI	(31.3–68.7)	(21.1–78.9)	(26.0–74.0)	(30.6–73.2)		(0.8–90.6)	(30.6–73.2)	(0.4–64.1)	(36.4–79.3)
*p*‐value		1.000		0.480		1.000		0.165	

^a^ The four cases lack of efficacy evaluation after treatment, so only 30 patients were included in the efficacy evaluation set (EES).

Abbreviations: BOR, best overall response; bTMB, blood tumor mutation burden; CI, confidence interval; CR, complete response; DCR, disease control rate; NE, not evaluable; ORR, objective response rate; PD, disease progression; PD‐L1, programmed death‐ligand 1; PR, partial response; SD, stable disease.

### 3.3 Biomarker analysis

High HDAC2 patients had a statistically longer mPFS compared with patients with low/medium HDAC2 (3.7 m vs. 1.4 m) (Figure 3D). But there are only two patients who expressed low/medium HDAC2 (6.67%, 2/30), so the difference was not significant (*p* = 0.106). Compared with positive PD‐L1, negative PD‐L1 patients had a slightly longer mPFS (3.7 m vs. 1.8 m, *p* = 0.350) (Figure 3E). However, the proportion of positive PD‐L1 patients was much lower than negative PD‐L1 (11.8% vs. 73.5%). Low‐level bTMB patients had a statistically longer mPFS compared to those with high‐level bTMB (3.8 m vs. 1.9 m, *p* = 0.110) (Figure 3F). ORR (9.1% vs. 0, *p* = 1.000) and DCR (59.1% vs. 16.7%, *p* = 0.165) were also numerically higher in low‐level bTMB group compared with high‐level bTMB group.

GEP analyses were performed in eight evaluable patients using gene signatures representing both immune cell and tumor characteristics. Two patient clusters were identified by unsupervised clustering, defined as “cold” and “hot” tumor microenvironments. Five patients were included in the “hot” cluster and three belonged to the “cold” cluster. Patients in both “cold” cluster and “hot” cluster can benefit from the study treatment. Nevertheless, “cold” tumor patients tend to benefit more from the study treatment (Figure S2).

### 3.4 Safety

No more than one patient experienced dose‐limiting toxicity during the first stage, so all 31 patients in the second stage received chidamide at the dose of 30 mg. Two patients discontinued chidamide (5.9%, 2/34) (owing to decreased platelet count and fever) and three discontinued envafolimab (8.8%, 3/34) (owing to decreased platelet count, fever, and simultaneously infective pneumonia and immune‐mediated lung disease).

The most common treatment emerging adverse events (TEAEs) were anemia (76.5%, 26/34), decreased platelet count (44.1%, 15/34), hypokalemia (41.2%, 14/34), decreased neutrophil count (38.2%, 13/34), and hypoalbuminemia (38.2%, 13/34) (Table 3). The most common TEAEs greater than or equal to Grade 3 were anemia (14.7%, 5/34), hypokalemia (11.8%, 4/34), decreased neutrophil count (8.8%, 3/34), and asthenia (8.8%, 3/34).

**TABLE 3:** Adverse events emerging during treatment.^a^

Adverse events	Any Grade No. (%)	≥Grade 3 No. (%)
Anemia	26 (76.5)	5 (14.7)
Decreased platelet count	15 (44.1)	1 (2.9)
Hypokalemia	14 (41.2)	4 (11.8)
Decreased neutrophil count	13 (38.2)	3 (8.8)
Hypoalbuminemia	13 (38.2)	0
Decreased white blood cell count	11 (32.4)	2 (5.9)
Decreased appetite	10 (29.4)	2 (5.9)
Hyponatremia	10 (29.4)	1 (2.9)
Fever	9 (26.5)	1 (2.9)
Loss of weight	9 (26.5)	0
Asthenia	8 (23.5)	3 (8.8)
Increased aspartate aminotransferase	8 (23.5)	0
Increased alanine aminotransferase	7 (20.6)	0
Decreased lymphocyte count	6 (17.6)	2 (5.9)
Arthralgia	6 (17.6)	1 (2.9)
Hyperglycemia	6 (17.6)	0
Hemoptysis	5 (14.7)	1 (2.9)
Hypocalcemia	5 (14.7)	0
COVID‐19	5 (14.7)	0
Dyspnea	5 (14.7)	0
Vomit	5 (14.7)	0
Pruritus	5 (14.7)	0
Sinus tachycardia	5 (14.7)	0
Hypothyroidism	5 (14.7)	0
Infective pneumonia	4 (11.8)	2 (5.9)
Rash	4 (11.8)	1 (2.9)
Upper respiratory tract infection	4 (11.8)	1 (2.9)
Increased serum creatine	4 (11.8)	0
Constipation	4 (11.8)	0
Albuminuria	4 (11.8)	0
Nausea	4 (11.8)	0
Abdominal pain	4 (11.8)	0
Cough	4 (11.8)	0
Dizziness	4 (11.8)	0
Peripheral edema	4 (11.8)	0

^a^ AEs occurring at any grade in more than 10% of patients are illustrated in this table.

## 4 DISCUSSION

For advanced NSCLC without driver mutation after resistance to ICI immunotherapy, Docetaxel single‐drug chemotherapy, with an ORR of about 10%,^11^ is usually recommended. In this phase II clinical trial, we expected an ORR of 24% for chidamide plus envafolimab. Among 34 patients enrolled in this trial, per‐protocol analysis of 30 patients showed that median follow‐up was 8.1 months. BOR of the two patients was PR. Unconfirmed ORR was 6.7%. The expected efficacy could not achieve after evaluation, so enrollment was terminated.

Definition of the resistance to ICI treatment comes from SITC.^23^ Under this definition, patients received ICI monotherapy. However, in this trial, 94.1% (32/34) of patients received combination therapy previously. For these patients, remission of disease may be induced by synergistic effect or independent cumulative drug effects of immunotherapy plus chemotherapy, making it difficult to attribute resistance to specific regimen.^25^ In this case, the definition of resistance was not suitable. That is, the actual proportion of primary resistance in this trial probably far exceeds the current statistical results of 26.5%. These patients respond badly to immune rechallenge therapy.^26^, ^27^ This may be a potential reason for the unsatisfactory ORR.

Biomarker analysis showed that ORR, DCR, and PFS of patients with high HDAC2 expression was numerically better than patients with low/medium HDAC2 expression, which was consistent with the mechanism of chidamide. Chidamide, functioning as an epigenetic modulator, selectively inhibits the activity of HDAC1, 2, 3, and 10, and performs its antitumor action via multiple mechanisms including inhibiting tumor cell cycles, inducing tumor cell apoptosis, and enhancing natural killer cells and antigen‐specific cytotoxic T cells‐mediated tumor killing.^28^ However, the number patients with low expression in HDAC2 was too small, so it is necessary to increase sample size to confirm the conclusion in the future.

Interestingly, the DCR and PFS of patients with negative PD‐L1 and low‐level bTMB expression were numerically better than those with positive PD‐L1 and high‐level bTMB. Previous studies have demonstrated that tumors can be divided into “hot tumors” characterized by high T cell infiltration, high PD‐L1 expression, and high TMB, as well as “cold tumors” characterized by low TMB, low MHC class I expression and low PD‐L1 expression.^29^, ^30^, ^31^ In contrast to “hot tumors” that are sensitive to ICI, “cold tumors” respond badly to ICIs monotherapy.^31^, ^32^, ^33^, ^34^ But our study found that chidamide plus envafolimab seems to have better efficacy in patients with “cold tumors.” This may be related to the epigenetic function of chidamide. Chidamide triggers chromatin remodeling by inhibiting the relevant HDAC isoform to increase the level of acetylation of chromatin histones, and thus produces epigenetic alterations, which in turn inhibits the tumor cell cycle and induces apoptosis. Chidamide, on the other hand, can also enhance immune cell‐mediated cytotoxicity. It activates immune cells such as peripheral lymphocytes to directly enhance their anti‐tumor effects and enhance lysis of tumor cell. Moreover, it can also up‐regulate the expression of tumor‐related antigens on the surface of tumor cells and activate natural killer (NK) cells or antigen‐specific CD8^+^ T cells to cause death of tumor cell.^13^, ^35^, ^36^ Using epigenetic antineoplastic drugs to enhance antigen expression and stimulate NK cells to kill tumor cells have also been shown to be closely related to the treatment of cold tumors.^37^, ^38^ This suggests that the combination of chidamide and envafolimab may have potential clinical efficacy in some anti‐PD‐1 resistant NSCLC patients, but further identification of this population (particularly negative PD‐L1/low‐level TMB/cold tumors) is needed.

Common adverse effects in this trial were hematological toxicity, including anemia, low platelet count, low neutrophil and white blood cell count. Most AEs were Grade 1–2. The adverse effects of Grade ≥3 were controllable, mainly anemia (14.7%), hypokalemia (11.8%), and decreased neutrophil count (8.8%), which were known common AEs of chidamide and envafolimab. Compared with the respective single drugs, the incidence of AEs of the combination therapy increased to a certain extent, possibly due to overlapping adverse effects of these two drugs.

The study was limited by the sample size. There were only two patients who achieved PR and two patients who expressed low/medium HDAC2. So it is necessary to increase sample size to confirm the conclusion in the future.

## 5 CONCLUSION

The combination of chidamide plus envafolimab showed efficacy signals in certain NSCLC patients after failure of anti‐PD‐1 treatment. The overall safety was controllable. But further identification of beneficial population is necessary to provide a basis for precision treatment.

## AUTHOR CONTRIBUTIONS

**Yaxiong Zhang:** Writing – original draft (equal); writing – review and editing (equal). **Zihong Chen:** Writing – original draft (equal); writing – review and editing (equal). **Yu Liu:** Writing – original draft (equal); writing – review and editing (equal). **Liang Han:** Investigation (equal); resources (equal); writing – review and editing (equal). **Wei Jiang:** Investigation (equal); resources (equal); writing – review and editing (equal). **Qiming Wang:** Investigation (equal); resources (equal); writing – review and editing (equal). **Jianhua Shi:** Investigation (equal); resources (equal); writing – review and editing (equal). **Liqin Lu:** Investigation (equal); resources (equal); writing – review and editing (equal). **Jianying Li:** Investigation (equal); resources (equal); writing – review and editing (equal). **Mingjun Zhang:** Investigation (equal); resources (equal); writing – review and editing (equal). **Yan Huang:** Investigation (equal); resources (equal); writing – review and editing (equal). **Yunpeng Yang:** Investigation (equal); resources (equal); writing – review and editing (equal). **xue Hou:** Investigation (equal); resources (equal); writing – review and editing (equal). **Li Zhang:** Conceptualization (equal); project administration (equal); resources (equal); supervision (equal); writing – review and editing (equal). **Jing Li:** Supervision (equal); writing – review and editing (equal). **Wenfeng Fang:** Conceptualization (equal); project administration (equal); resources (equal); supervision (equal); writing – review and editing (equal). **Gang Chen:** Investigation (equal); project administration (equal); resources (equal); supervision (equal); writing – review and editing (equal).

## FUNDING INFORMATION

This work was supported by Chinese National Natural Science Foundation project (Grant No. 82102872, 82173101, 82241232, 82272789), Guangzhou Science and Technology Program (Grant No. 202002020074) and Guangdong Basic and Applied Basic Research Foundation (Grant No. 2022A1515011873).

## CONFLICT OF INTEREST STATEMENT

The authors have no financial or relationship conflicts to disclose.

## ETHICS SATATEMENT

This study was approved by the Institutional Review Board (IRB) of Sun Yat‐Sen University Cancer Center and with a written informed consent obtained from all patients.

## Supporting information


Figure S1.

Figure S2.

Table S1.

## Data Availability

The datasets used and/or analyzed during the current study are available from the corresponding author on reasonable request.
