# Supplementary figures and images for: Chidamide plus envafolimab as subsequent treatment in advanced non‐small cell lung cancer patients resistant to anti‐PD‐1 therapy: A multicohort, open‐label, phase II trial with biomarker analysis
Source: Cancer Med. 2024 Apr 10;13(7):e7175. doi: 10.1002/cam4.7175 (PMC11004905; doi:10.1002/cam4.7175)

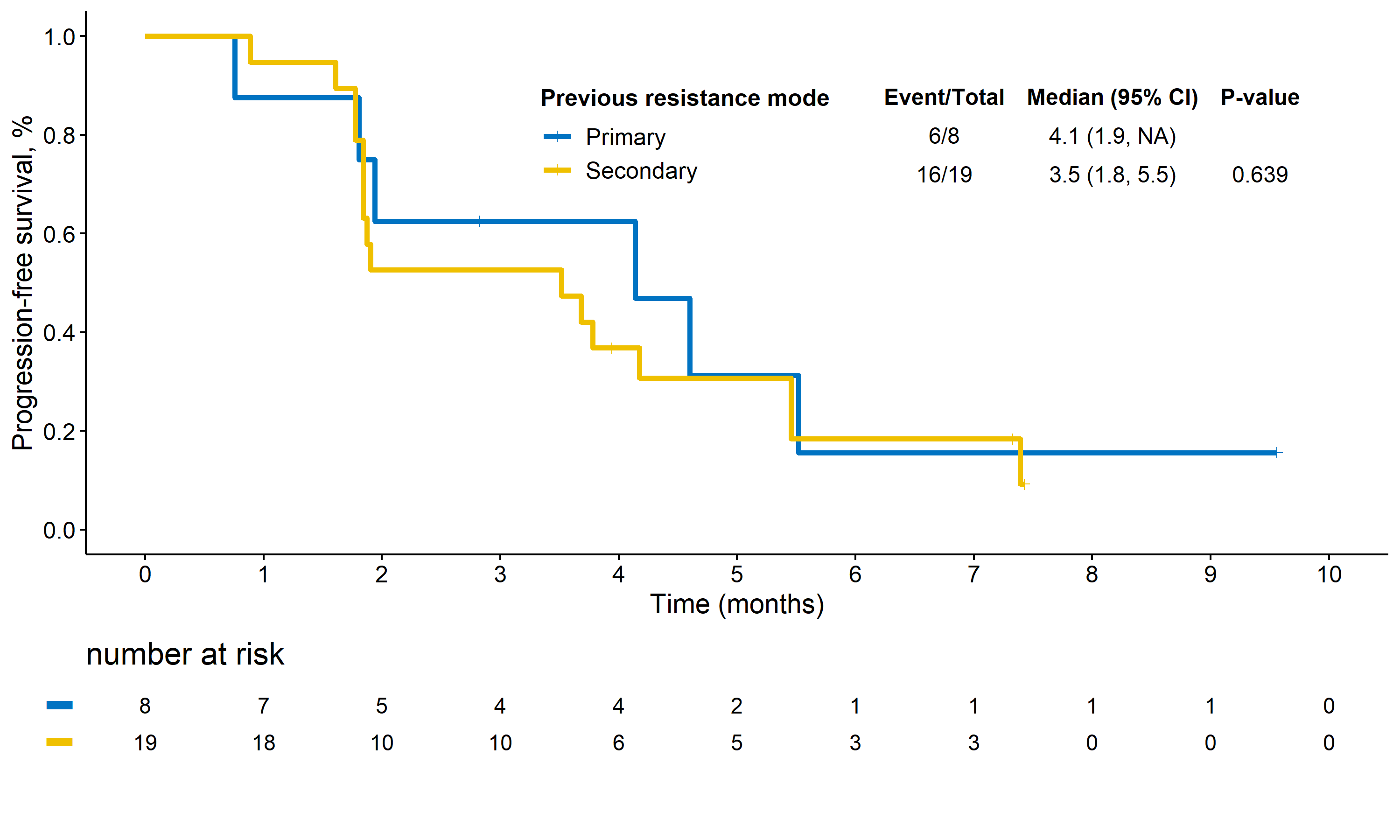


**Supplementary Fig. 1 PFS** **by resistant type (primary resistant versus secondary resistant).**

Supplement: Supplementary file 1 — Figure S1. [file CAM4-13-e7175-s002.docx]
