# Supplementary material for: Chidamide plus envafolimab as subsequent treatment in advanced non‐small cell lung cancer patients resistant to anti‐PD‐1 therapy: A multicohort, open‐label, phase II trial with biomarker analysis
Source: Cancer Med. 2024 Apr 10;13(7):e7175. doi: 10.1002/cam4.7175 (PMC11004905; doi:10.1002/cam4.7175)
Supplement: Supplementary file 2 — Figure S2. [file CAM4-13-e7175-s003.docx]

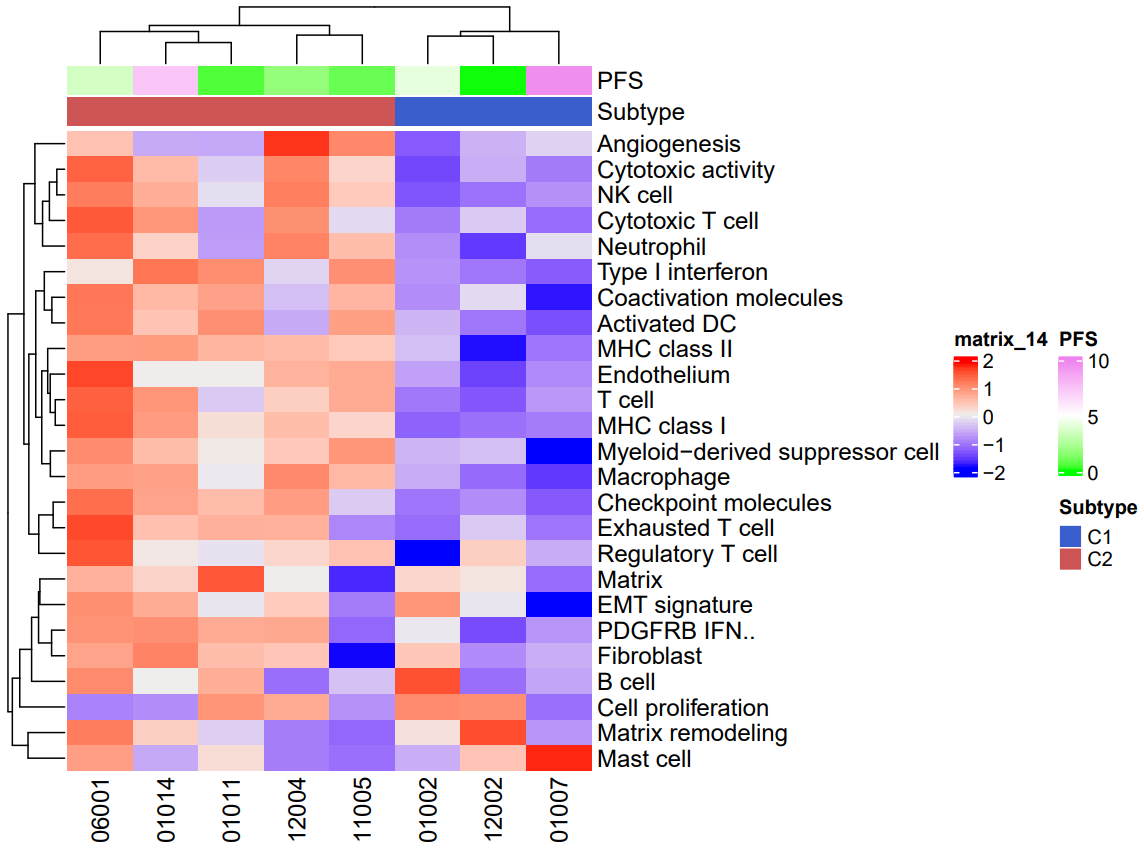


**Supplementary Fig. 2 Gene expression signatures in tumor microenvironment and association with PFS.**
