# Supplementary material for: Chidamide plus envafolimab as subsequent treatment in advanced non‐small cell lung cancer patients resistant to anti‐PD‐1 therapy: A multicohort, open‐label, phase II trial with biomarker analysis
Source: Cancer Med. 2024 Apr 10;13(7):e7175. doi: 10.1002/cam4.7175 (PMC11004905; doi:10.1002/cam4.7175)
Supplement: Supplementary file 3 — Table S1. [file CAM4-13-e7175-s001.docx]

**Supplementary Table 1. Detailed information of tested genes and its classification.**

| **Classification** | **Gene** | | | | | | | |
| --- | --- | --- | --- | --- | --- | --- | --- | --- |
| T cell | CD3D | CD3E | CD3G | CD247 | CD2 | CD6 |  |  |
| B cell | CD19 | CD20 | CD79A | CD79B | CXCR5 |  |  |  |
| Cytotoxic activity | PRF1 | GZMA | GZMB | GZMH | NKG7 |  |  |  |
| Macrophage | CD68 | CD163 | CSF1R | FCGR1A | FCGR1B |  |  |  |
| Fibroblast | ACTA2 | POSTN | FAP | COL1A1 | COL1A2 |  |  |  |
| PDGFRB IFNγ | IFNG | CXCL9 | CXCL10 | CXCL11 |  |  |  |  |
| Type I interferon | MX1 | BST2 | MX2 | OAS1 | OASL | OAS3 | ISG15 |  |
| Cell proliferation | MKI67E2F1 | MYBL2 | BUB1 | PLK1 |  |  |  |  |
| Regulatory T cell | FOXP3 | IL2RA | KLF2 |  |  |  |  |  |
| Neutrophil | ALOX15B | ALOX5 | CA4 | CREB5 | CSF3R | CXCR1 | CXCR2 | FCAR |
|  | FPR1 | FPR2 | LILRB2 | MME | S100A12 | SIGLEC5 | TNFRSF10C | |
| MHC class I | HLA-A | HLA-B | HLA-C | HLA-E | HLA-F | HLA-G | MR1 |  |
| MHC class II | CD74 | HLA-DMA | HLA-DMB | HLA-DOA | HLA-DOB | HLA-DPA1 | HLA-DPB1 | |
| Mast cell | CEACAM8 | CMA1 | CPA3 | CTSG | GATA2 | HDC | KIT | MPO |
|  | MS4A2 | PRG2 | PTGS1 | TAL1 | TPSAB1 |  |  |  |
| Myeloid-derived  suppressor cell | ANPEP | ARG1 | CD33 | CD34 | FUT4 | IL10 | ITGAM | TGFB1 |
| Exhausted T cell | CD274 | CD276 | CD8A | LAG3 | PDCD1LG2 | TIGIT |  |  |
| Cytotoxic T cell | CD8A | CD8B | GZMA | GZMB | PRF1 |  |  |  |
| Matrix | FN1 | COL1A1 | COL1A2 | COL4A1 | COL3A1 | VTN | LGALS7 | LGALS9 |
|  | LAMA3 | LAMB3 | LAMC2 | TNC | ELN | COL5A1 | COL11A1 | |
| Angiogenesis | VEGFA | VEGFB | VEGFC | PDGFC | CXCL8 | CXCR2 | FLT1 | PGF |
|  | CXCL5 | KDR | ANGPT1 | ANGPT2 | TEK | VWF | CDH5 |  |
| Endothelium | NOS3 | KDR | FLT1 | VCAM1 | VWF | CDH5 | MMRN1 | ENG |
| **Classification** | **Gene** | | | | | | | |
| Endothelium | CLEC14A | MMRN2 |  |  |  |  |  |  |
| EMT signature | SNAI1 | SNAI2 | TWIST1 | TWIST2 | ZEB1 | ZEB2 | CDH2 |  |
| Matrix remodeling | CA9 | MMP9 | MMP2 | MMP1 | MMP3 | MMP12 | MMP7 | MMP11 |
|  | PLOD2 | ADAMTS4 | ADAMTS5 | LOX |  |  |  |  |
| Checkpoint molecules | PDCD1 | CD274 | CTLA4 | LAG3 | PDCD1LG2 | BTLA | HAVCR2 | TIGIT |
|  | VSIR | C10orf54 |  |  |  |  |  |  |
| Coactivation molecules | CD28 | CD40 | NFRSF4 | ICOS | TNFRSF9 | CD27 | CD80 | CD86 |
|  | CD40LG | CD83 | TNFSF4 | ICOSLG | TNFSF9 | CD70 |  |  |
| Activated DC | LAMP3 | CD80 | CD83 | CD40 | CCR7 | FSCN1 | CCL17 | CCL19 |
|  | CCL22 | CD86 | CCR7 | CCL8 |  |  |  |  |
